# Endogenous Retroviruses Walk a Fine Line between Priming and Silencing

**DOI:** 10.3390/v12080792

**Published:** 2020-07-23

**Authors:** Harrison Cullen, Andrea J. Schorn

**Affiliations:** Cold Spring Harbor Laboratory, Cold Spring Harbor, NY 11724, USA; cullen@cshl.edu

**Keywords:** endogenous retrovirus (ERV), tRNA-fragment (tRF), small RNA silencing, tRNA primer binding site (PBS), RNA interference (RNAi), intracisternal A-particle (IAP), early transposon (ETn), human immunodeficiency virus (HIV)

## Abstract

Endogenous retroviruses (ERVs) in mammals are closely related to infectious retroviruses and utilize host tRNAs as a primer for reverse transcription and replication, a hallmark of long terminal repeat (LTR) retroelements. Their dependency on tRNA makes these elements vulnerable to targeting by small RNAs derived from the 3′-end of mature tRNAs (3′-tRFs), which are highly expressed during epigenetic reprogramming and potentially protect many tissues in eukaryotes. Here, we review some key functions of ERV reprogramming during mouse and human development and discuss how small RNA-mediated silencing maintains genome stability when ERVs are temporarily released from heterochromatin repression. In particular, we take a closer look at the tRNA primer binding sites (PBS) of two highly active ERV families in mice and their sequence variation that is shaped by the conflict of successful tRNA priming for replication versus evasion of silencing by 3′-tRFs.

## 1. Introduction

Reverse transcription and long terminal repeat (LTR) retroelements are ancient components of eukaryotic genomes [1]. In fact, reverse transcriptase (RT) is one of the most abundant genes in organisms with high copy numbers of retroelements such as mammals [1,2,3]. LTR-retroviruses encode envelope proteins to form virus particles and infect neighboring cells or other organisms, while LTR-retrotransposons that lack functional envelope proteins replicate within viral-like particles (VLPs) to integrate into the same cell. The majority of LTR-retrotransposons in mammals are closely related to known infectious LTR-retroviruses and are therefore called endogenous retroviruses (ERVs). Based on the phylogenetic relationship of their RT genes, mammalian ERVs belong to the *Retroviridae* genus, while LTR-retrotransposons prevalent in other phyla such as the Gypsy and Copia superfamilies are *Metaviridae* and *Pseudoviridae*, respectively [4,5,6]. All three genera include infectious, viral elements with an envelope gene as well as endogenous transposons that proliferate in a strictly intracellular fashion. Endogenous LTR-retrotransposons that lost a functional envelope gene are inherited vertically but may in principle, at low frequency, spread to other organisms by horizontal transfer, a process used by all transposable elements to enter new host species [7,8]. ERVs have become resident aliens in mammalian genomes and many of them were co-opted by their hosts to fulfill essential cellular functions, for example, during placentation and imprinting [9,10,11]. ERV promoter and enhancer activities as well as their protein domains have been useful building blocks during evolution, while their repetitive ends induce recombination and mobility of intact, full-length elements is highly mutagenic [12,13,14,15,16,17]. Hence, their expression needs to be carefully monitored by the cell. This review will discuss how small RNAs identify and silence ERVs when they are released from heterochromatin during epigenetic reprogramming.

**Figure 1:**
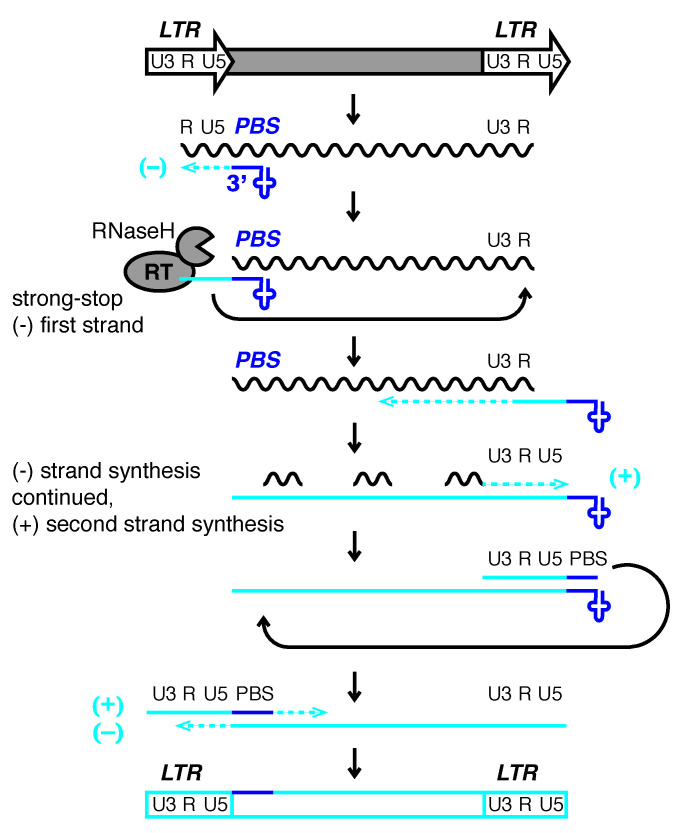
Model of reverse transcription of long terminal repeat (LTR)-retrotransposons and -viruses. LTRs encode promoter elements and termination signals. The RNA transcript contains a region repeated at either end (R), a 5′ unique segment (U5), and a segment only included at the 3′-end of the RNA (U3). The 3′-end of cellular tRNAs (blue cloverleaf) primes reverse transcription by hybridizing to the primer binding site (PBS). While this segment is being copied into first-strand cDNA (light blue line), also called minus (−) strong stop DNA, the RNaseH activity of reverse transcriptase (RT) degrades the template RNA. The elongating cDNA is transferred to the 3′-end of the retrotransposon transcript hybridizing to the R region. The remaining RNA is partially degraded by RNaseH leaving behind primers for second-strand, plus (+) cDNA synthesis. In *Retroviridae*, the plus strand PBS is a copy of the tRNA primer, while the minus strand is a copy of the original PBS sequence. After another transfer event, first (−) and second (+) strand synthesis are completed to result in a full-length, double-stranded retroviral DNA that will be integrated into the host genome.

With few exceptions, LTR-retroelements use host tRNA to prime reverse transcription and copy their RNA into DNA for insertion into the genome (Figure 1) [18,19,20,21]. tRNAs are essential host molecules that are abundantly available at the point that viral proteins are translated. Retroviral proteins bind specific tRNAs with high affinity and recruit them to the virus particle or VLP where their 3′-end initiates reverse transcription at the tRNA primer binding site (PBS). Small RNAs derived from the 3′-end of tRNAs (3′-tRFs) target LTR-retroelements at the PBS and control their mobility and expression [22,23,24]. These highly conserved sequence motifs are a prerequisite for replication and allow host defense mechanisms to identify active LTR-retroelements. ERV sequences make up ~10% of the mouse and human genome, but only a few full-length copies are capable of retrotransposition. Variations of the PBS sequences amongst two highly active ERV families hint at what may be the perfect recipe for fitness—mutations that reduce tRF-inhibition but still allow tRNA binding and priming.

## 2. Epigenetic Reprogramming of ERVs

### 2.1. De-repression of ERVs during Chromatin Reprogramming

ERVs are usually embedded in repressive heterochromatin, but importantly become active during epigenetic reprogramming in development and disease. Mammals undergo genome-wide epigenetic reprogramming in the embryo right after fertilization and in the germline to obtain totipotency and set aside cells for the next generation [25,26,27]. ERV transcription is repressed by DNA and histone methylation. The histone methyltransferases G9a/GLP, SETDB1, EZH2, histone demethylase KDM1A, as well as the de novo DNA methyltransferases DNMT3a/b, DNMT3L, and DNMT3C establish heterochromatin at different classes of ERVs as discussed in detail elsewhere [28,29]. DNA methylation status can directly correlate with ERV transcription [29,30,31,32,33]. However, absence of DNA methylation does not necessarily lead to ERV expression, as long as histone H3 lysine K9 tri-methylation (H3K9me3) can be maintained [34,35,36,37]. The histone H3K9me3 methyltransferase SETDB1 is acting in complex with KAP1/TRIM28 on fully methylated or fully unmethylated DNA, but not hemi-methylated DNA that is occupied by NP95 [37]. This finding resolves why ERV expression is not always observed in stable methylation knock-outs but is observed in inducible knock-outs that undergo temporary hemi-methylation, and most importantly during epigenetic reprogramming in vivo which includes a hemi-methylated state [32,33,37]. Like any gene, transposon expression depends on multiple layers of repressive and permissive control on the RNA, DNA, and protein level. Removal of silent chromatin marks allows transcription factors (TFs) to bind DNA and promote or inhibit ERV transcription [38,39,40]. LTR sequences, for example, contain species-specific TF binding sites that promote temporary expression of ERVs and neighboring genomic sequences during development [38]. After reprogramming, chromatin patterns at transposable elements need to be re-established through DNA and RNA recognition. KRAB zinc finger proteins (ZFPs) have co-evolved with their transposon targets and guide heterochromatin formation by SETDB1, TRIM28/KAP1 through binding to highly conserved DNA sequence motifs in ERVs [41]. For example, some KRAB-ZFPs bind to the PBS of select ERVs or the polypurine tract that primes second strand reverse transcription during ERV replication [42]. Once transcribed, ERV expression, translation, and reverse transcription must be restricted by the cell, and small RNAs have the ability to recognize and target transposon RNA for silencing.

### 2.2. ERVs as Epigenetic Switches in Development and Disease

The propensity of ERVs to attract diverse silencing machineries that act upon specific transposon families at different stages of development make them ideal epigenetic switches [17]. An estimated 6–30% of transcripts in mouse and human embryonic and somatic tissues are driven by retrotransposon promoters in a highly tissue-specific manner [43]. ERV families define gene-regulatory networks throughout development [12,13]. Transcription of murine MERV-L elements marks the totipotent two-cell stage in early embryos [44]. Human HERV-H expression is indicative of the naive embryonic stem cell state and essential for pluripotency [45,46]. In addition, ERV LTR promoter-enhancer activity drives non-coding, stem-cell specific transcripts that maintain the undifferentiated state and are crucial for cell identity [47,48,49,50]. More than 800 LTRs from the ERV-L and mammalian apparent LTR-retrotransposon (MaLR) families act as alternative promoters and first exons to drive stage-specific gene expression in mammalian oocytes and the developing zygote [25,51]. Taken together, temporary release of transposon silencing during reprogramming affects the transcriptome through (i) expression of potentially mobile, mutagenic, intact transposons, (ii) expression of transposon-derived, long non-coding regulatory RNAs (lncRNAs), and (iii) expression of neighboring genes or lncRNAs driven by promoter-enhancer activities of the LTRs.

The epigenetic state of ERVs and transposable elements in general can not only lead to developmental stage- and cell-type-specific expression but also establish epigenetic alleles or “epialleles” that result in differential expression between isogenic offspring [52,53]. Epialleles can be stable and inherited if they entirely escape reprogramming or “metastable” and lead to stochastic changes of the epigenetic state in the offspring [53]. The most famous example of an ERV-induced metastable epiallele is the differential methylation of an intracisternal A-particle (IAP) insertion upstream the mouse Agouti gene which results in varying fur color and obesity in siblings [30]. In fact, such metastable epialleles of IAP are extremely abundant genome-wide, but few of them affect neighboring gene expression [31]. Select ERVs, particularly a set of IAP elements, are protected from reprogramming in the early embryo and the germline, and therefore inherit their epigenetic state as stable epialleles [54,55]. Human ERV (HERV) methylation varies between individuals that could be metastable epialleles, but it is hard to exclude genetic variation [53,56]. Notably, many imprinted genes are derived from LTR-retrotransposons. Imprinted genes of the sushi-ichi-related retrotransposon homologs (SIRH) are common to placental mammals and derived from *Metaviridae* gypsy-elements [57]. Lineage-specific *Retroviridae* ERV insertions mediate imprint establishment at murine loci such as retrotransposon-like 1 (*Rtl1*), *Rasgrf1*, *Impact*, and *Slc38a4* [58,59,60]. The murine ERVK family drives non-canonical, histone-dependent imprinting in the extraembryonic lineage [61]. Imprinted loci are established during epigenetic reprogramming of the germline and persist in the early embryo [54,62,63]. In contrast to epialleles, heterochromatin induction at imprinted loci is not stochastic but established at either the paternal or maternal allele, respectively, and is essential for proper development.

Similar to epigenetic reprogramming in development, ERV reactivation has been observed in other tissues with high epigenetic plasticity, particularly in the course of disease [17,64,65]. The role of ERVs in cancer extends beyond their value as diagnostic markers for aberrant reprogramming. They are frequently epigenetically reactivated as cryptic promoters in cancer and drive oncogene expression [47,66,67,68]. Indeed, LTR-retroviruses were originally identified as the causative agents of transmissible tumors in chicken, mice, and humans [69]. Those ‘RNA tumor viruses’ include Rous sarcoma virus (RSV), mouse mammary tumor virus (MMTV), and human T cell leukemia virus 1 (HTLV-1). However, expression of endogenous HERV proteins can also tip the scales and trigger an immune response that drives tumor cells into apoptosis [70].

## 3. Small RNA Silencing of ERVs

### 3.1. Small RNAs during Reprogramming in the Mammalian Embryo

When transposable elements are released from repressive chromatin during epigenetic reprogramming in development and disease, small RNA-mediated silencing mechanisms become crucial to limit transposition and maintain genome integrity [71,72,73]. Argonaute (AGO) and P-element induced wimpy testis (PIWI) proteins are the core of the RNA interference (RNAi) machinery and bind small RNAs to mediate silencing of complementary sequences. Genome-wide epigenetic reprogramming in mammals invokes transposon expression in the zygote right after fertilization and in developing germ cells which determine transposon burden of the next generation [25,26,27]. By far the most is known about PIWI-interacting RNAs (piRNAs) silencing ERVs in the male germline and there are many excellent reviews on the topic [72,73,74]. Pre-pachytene stage piRNAs in primordial germ cells of mice are highly enriched in transposon sequences and inhibit ERVs post-transcriptionally through mRNA binding as well as transcriptionally by guiding de novo methylation [75,76,77]. Transcriptional silencing guided by small RNAs is well established in other eukaryotes, yet, how PIWI proteins recruit the chromatin machinery to transposon sequences in mice remains elusive [73,78]. Small RNA-mediated silencing does not only prevent mutagenic damage from transposition, but importantly regulates repetitive elements that have been co-opted by the host to serve essential functions. For example, silencing of the paternally imprinted *Rasgrf1* locus in mouse is mediated by piRNAs that target an ERV sequence [60].

In the female germline of *Muridae*, endogenous small interfering RNAs (endo-siRNAs) target transposon mRNA and protect oocytes [26,79,80,81]. An oocyte-specific isoform of the endonuclease DICER is produced due to temporary reactivation of an intronic ERV and processes endo-siRNAs from long double-stranded RNAs which invoke an interferon immune-response in most other cell types of mammals [82]. Human oocytes express a *PIWI* gene that is lacking in several rodents and produce “oocyte short piRNAs” that target HERVs [83]. However, deletion of DICER and PIWI proteins results in chromosomal defects that cannot be explained by control of active transposition alone but point to a role of the RNAi machinery more broadly in repeat and genome stability [84]. Hundreds of DICER-dependent miRNAs originate from and theoretically target ERV sequences in mice and humans [85,86,87]. A functional relationship has yet been shown for one miRNA that regulates the *Rtl1* imprinted gene in mouse placenta [59]. MiRNAs target and sense LTR-retroelements in plant development [88,89] and regulate non-LTR retroelements such as long interspersed nuclear element (LINE)-1 in human [90,91]. However, the impact of miRNAs on genome-wide ERV reactivation in mammals is unclear.

Small RNAs expressed in the germline are well-known to restrict transposable elements, but it is less clear how transposition is avoided during the first wave of reprogramming that enables totipotency in the pre-implantation embryo. DICER-dependent silencing of IAP and MERV-L transcripts was detected up to the eight-cell stage in mouse embryos [92]. However, siRNAs and piRNAs abundant in gametes are depleted at later stages of pre-implantation with minimal heterochromatin and absent in other somatic tissues that reactivate transposable elements in mouse and human [26,79,80,81,93]. ERVs are strongly expressed in the absence of H3K9me3 in mouse embryonic stem cells (mESCs) and preimplantation embryos [25,35,94]. Indeed, one of the most mutagenic transposon families in mouse was coined “Early Transposon” (ETn) because of its strong expression in early embryogenesis [95]. Small RNAs derived from the 3′-end of mature tRNAs (3′-tRFs) are expressed in stem cells and tissues of pre-implantation mouse embryos as well as cancer cells with high ERV burden and strongly inhibit retrotransposition (our unpublished results and [22]). 3′-tRFs include the post-transcriptional trinucleotide CCA-tail of mature tRNAs and consequently do not match genomic tDNA sequences but perfectly match LTR-retroelements at their highly conserved PBS. Hence, 3′-tRFs recognize any potentially mobile ERV that is able to bind host tRNA for replication, thus distinguishing harmful from harmless copies amongst large numbers of ERV-derived sequences [20].

### 3.2. tRNA Fragments and RNA Interference

tRFs are a novel class of small non-coding, regulatory RNAs with distinct types, length, and diverse biological functions: 3′-tRFs and 5′-tRFs that are not the reverse complement of each other, stress-induced tRNA halves, internal fragments, and tRFs of precursor tRNAs [20,96]. Several tRF types bind to AGO and PIWI proteins in multiple organisms [23,24,97,98,99,100,101,102,103], and can guide silencing of reporter constructs with complementary binding sites in their 3′ untranslated region (UTR) [98,102,104]. In fact, some previously annotated miRNAs turn out to be tRFs [101,105,106,107]. Recently, biological targets of 3′-tRFs were uncovered and post-transcriptional gene silencing was confirmed [22,101]. Due to their perfect sequence complementarity to the PBS of LTR-retroelements, 3′-tRFs have tens of thousands of ERV targets in mammalian genomes. 3′-tRFs come in two distinct sizes that are expressed in cell type specific ratios: 17–19 nucleotides (nt) long tRF3a fragments specifically interfere with reverse transcription, while 22 nt tRF3b fragments inhibit coding-competent ERVs with all the hallmarks of miRNA silencing [22]. Indeed, a glycine tRF3b fragment has been shown to act as an AGO2-dependent miRNA in vivo targeting an essential, human replication protein [101]. tRF3b fragments that target the PBS in the 5′-UTR of ERVs decrease RNA and protein levels [22]. Interestingly, the majority of 3′-tRFs bound to endogenous AGO2 in human cells are the shorter tRF3a fragments [103], which are also the major, functional cargo of the *Tetrahymena* PIWI protein Twi12 [99], suggesting 3′-tRFs are highly conserved substrates of the RNAi silencing machinery. tRNAs are thought to have evolved from minihelices including the entire portion of 3′-tRFs and a few nucleotides of the ‘modern day’ double helix 5′-end [108]. Minihelices are functional substrates for the CCA-adding enzyme during tRNA maturation [109], and specific nucleotides in the acceptor stem portion of the minihelix are necessary and sufficient for aminoacylation, supporting the idea of a primordial code by tRNA minihelices [108,110]. The L-shaped tertiary structure of tRNA resembles other double-stranded RNA substrates of RNAi. The presence of 3′-tRFs in AGO/PIWI pulldowns, despite large amounts of miRNAs or piRNAs in some of those cell types, argues that they are functional substrates and direct the RNAi machinery to transposon targets [20].

Given the variable expression of ERVs throughout development and even between genetically identical individuals, small RNAs arguably serve well to sense and adjust ERV expression. Small RNA mediated host defense often scales with transposable element burden and acts at different levels: piRNAs in mammals are produced from single-stranded precursor RNA of clusters of transposon sequences already in the host genome, and siRNAs are produced from double-stranded RNA of transposon transcripts [73,82,111,112]. 3′-tRFs can be seen as an innate immune response— they readily exist in all organisms without prior exposure to that particular LTR-retroelement and specifically recognize any potentially mobile copy by the presence of its conserved PBS. In this sense, the PBS is the Achilles’ heel of retrotransposition: if this sequence is mutated, the transposon evades tRF silencing but also loses its ability to replicate using host tRNAs. tRF targeting is likely a highly conserved mechanism of small RNA-mediated transposon control. While many small RNAs also target mRNA of domesticated transposon domains, 3′-tRFs enable the host to discriminate self from non-self: transposon-derived coding sequences with no PBS go incognito, while mobile elements and active retroviruses require an intact PBS sequence, with complementarity to 3′-tRFs.

## 4. Two Highly Active ERV Families in Mouse and Their Primer Binding Site Variations

### 4.1. Replication of ERVs and other Retroviridae

Genome sequencing projects have given insight into the spread of repetitive elements over evolutionary time and into which transposons cause polymorphisms when comparing strains or individuals. In mice, ERVs are highly active causing an estimated 10% of germline mutations in today’s inbred laboratory strains [14,113]. The ERV superfamilies IAP (*Gammaretroviridae*) and ETn/MusD (*Betaretroviridae*) are among the most active transposons in mouse with the majority of novel insertions being non-autonomous copies carried over by a few coding family members [14,114,115]. ETn elements are non-autonomous and depend on the enzymatic machinery of autonomous MusD elements related by identical LTR sequences and usage of the same primer tRNA. Autonomous ERVs contain at least three open reading frames and undergo mRNA splicing and translation to produce the group-specific-antigen (Gag) polyprotein that assembles the VLP, protease (Pr) for maturation of the gene products, and reverse transcriptase (RT) [20,116]. Two copies of unspliced viral template RNA are recruited directly into the VLP for reverse transcription. Non-autonomous elements are non-coding and hitchhike on the enzymatic machinery provided by the autonomous family member. Their genomic RNA does not undergo splicing or translation but is directly transported into the VLP. From studies of murine leukemia virus (MLV), human immunodeficiency virus (HIV), and RSV, we have an idea about RNA content and stoichiometries in the VLP or virus particle [117,118]. The majority of RNA molecules are tRNAs with estimates ranging from 70–700 tRNAs per particle. Interestingly, other co-packed RNAs (i.e., 7SL RNA, Y RNAs, U6 snRNAs), although not abundant, are transcripts generated by polymerase III (POLIII), just like tRNAs, suggesting an intersection of POLIII-regulated transcripts with retroelement evolution. Non-LTR retroelements are thought to be evolutionarily older and likewise interact with POLIII transcripts: human LINEs frequently pick up U6 RNA during retrotransposition [119] and have reverse transcribed 7SL RNA to generate Alu elements in *Xenopus*, *Drosophila*, and human [120].

The most highly enriched molecules in viral particles or VLPs are the tRNA primer isotype used for reverse transcription, for example, lysine for HIV-1, representing 70% of all packaged tRNAs [118]. IAP elements use phenylalanine tRNA (coding the GAA triplet) to prime reverse transcription and are inhibited by 3′-fragments of those same tRNAs (3′-tRF^Phe-GAA^) [22]. ETn/MusD elements use the same primer as HIV, tRNA^Lys-UUU^, and are targeted by 3′-tRF^Lys-UUU^ [22,24]. tRNAs with a specific codon can include different isodecoders with variable sequences, indicated by numbers in addition to the codon triplet letters [110,121], so more precisely ETn/MusD and HIV are primed by tRNA Lysine^3^-UUU and inhibitory 3′-tRFs are derived from this exact sequence. HIV and MLV only use cytoplasmic tRNAs that have passed rigorous quality control by the cell and are capable of being charged with the correct amino acid [122]. Primer tRNAs are enriched in viral particles by RT in several *Retroviridae* such as HIV, RSV, MLV, and avian sarcoma leukosis virus (ASLV) [123]. In the case of HIV, Gag-Pol proteins recruit the Lysine^3^-UUU tRNA primer through interaction with Lysyl-tRNA synthetase and prevent aminoacylation of the tRNA [124]. Some *Retroviridae* like RSV follow this route, while others like MLV do not package tRNA synthetases [123,124]. In general, RT enzymes evolved to bind specific tRNAs with high affinity to initiate reverse transcription. tRNA priming requires more than an 18 nt match at the PBS and tRNA annealing in HIV-1 is concomitant with a series of conformational changes of the retrovirus RNA during VLP formation [125]. Although short oligonucleotides prime reverse transcription of heat-denatured templates in vitro, priming and elongation in vivo require the interaction of the RT enzyme with the full-length, structured tRNA and mutations at critical residues outside the 3′-acceptor arm impair processivity [18,126]. This results in a de facto suppression of reverse transcription in vivo when tRNAs and tRFs compete [22,24].

### 4.2. Measuring LTR-Retrotransposon Activity

There are a number of techniques to quantify active retrotransposition and probe its regulation. RNA levels are often used as a proxy for transposon activity, but transcription is only one step in the life cycle of transposons and does not necessarily reflect mobility and mutagenic burden, for example, if transposition is inhibited post-transcriptionally by small RNAs. LTR-retrotransposon activity can be experimentally assessed almost every step of the way. Capped, spliced RNA levels reflect mRNA before translation [43,48,127], while unspliced full-length transcripts are the viral “genomic” RNA template for reverse transcription. RNaseH intermediates of strong stop, first strand cDNA can be detected by 5′-RACE of uncapped RNA ends using transposon-specific primers [22]. Extrachromosomal viral DNA copies can be separated from genomic host DNA by a simple alkaline-lysis. Viral particles and VLPs have been purified to sequence packaged RNA or retroviral DNA of reverse transcription intermediates [117,128]. These DNA intermediates can be functional and subsequently integrate but also reveal aberrant products of transposons that lack the ability to integrate. Extrachromosomal cDNA of LTR-retroelements that persists as episomal circular DNA is generally considered a dead-end abrogation product but can be actively transcribed and contribute to the viral protein load in the cell [129]. Ultimately, retrotransposition assays with reporter gene cassettes allow to quantify successful integration and to trace intermediates of a specific element [130]. Transposition assays are typically performed in a naive host such as in human cells for murine ERVs, because the original host contains multiple layers of defense mechanisms and thousands of untagged, related transposon copies that may be co-mobilized and confound quantification.

Retrotransposition assays with an autonomous and a non-autonomous element allow to dissect the effect of PBS mutations and endogenous 3′-tRFs on ERV activity. A coding-competent MusD element with a scrambled PBS can no longer prime its own reverse transcription but can still replicate its non-autonomous partner, ETn [22]. Without a functional PBS, MusD escapes targeting by tRFs resulting in increased expression of its enzymatic machinery that mediates increased retrotransposition of ETn. Disruption of the PBS of the non-autonomous ETn simply results in a “dead” copy. Hence, coding elements with mutations that prevent tRF silencing and mobility can be potent sources of viral protein production and be able to retrotranspose non-coding elements, as long as the latter retain a functional PBS. That applies to elements such as MusD that are able to efficiently mobilize non-autonomous copies *in trans*, and perhaps explains the much higher copy numbers for ETn over MusD in murine genomes. ERVs with *cis*-preference, such as IAP elements, bind and retrotranspose their own RNA *in cis,* at much higher frequencies than RNA from other elements [131]. For such retroelements, disruption of tRNA priming should immediately penalize retrotransposition. Retrotransposition assays, Gag protein and mRNA detection, together with ERV-specific 5′-RACE of uncapped retroviral RNA intermediates have revealed mechanisms of tRF inhibition: tRF3a fragments decrease RNaseH cleavage products around the PBS, an indicator of successful priming and processivity by RT, while tRF3b fragments reduce mRNA and protein levels similar to miRNAs [22]. Therefore, autonomous elements are strongly affected by both types of tRFs, while non-coding elements are regulated by tRF3a fragments only at the RT step.

### 4.3. A Trade-Off between Priming and Silencing?

**Figure 2:**
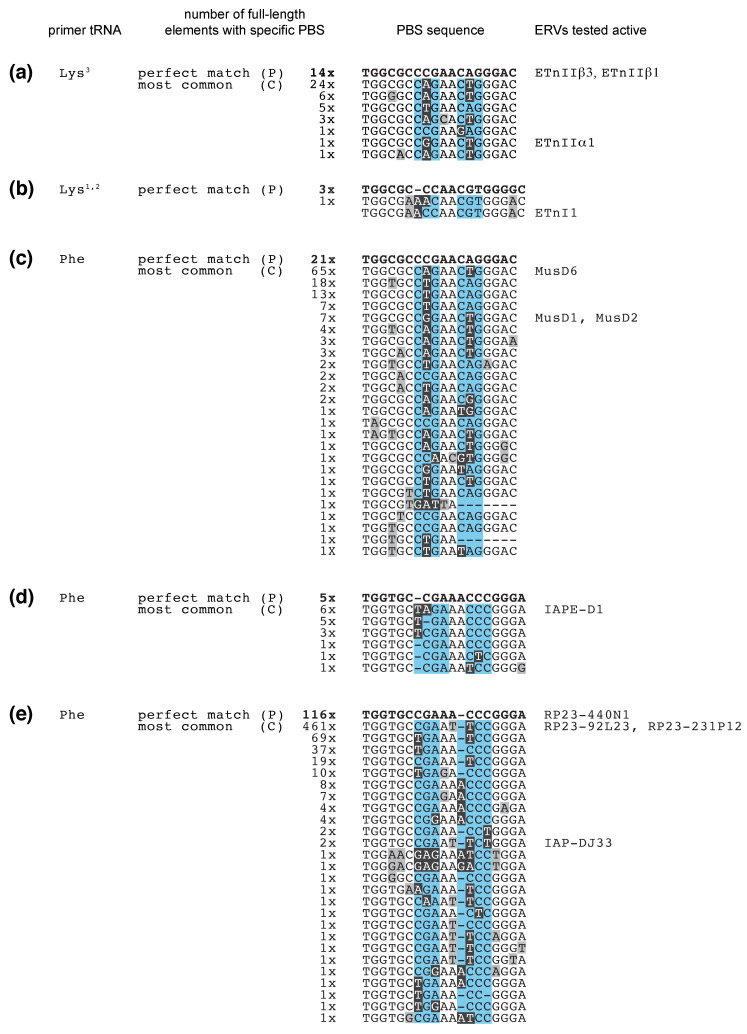
Sequence variation in the 18 nucleotide tRNA primer binding site (PBS) of the murine endogenous retroviruses (ERVs) (**a**) ETnIIbeta (tRNA^Lys3^), (**b**) ETnIIbeta (tRNA^Lys1,2^), (**c**) MusD (tRNA^Lys3^), (**d**) IAPE (tRNA^Phe^), and (**e**) IAP (tRNA^Phe^). The PBS sequences with perfect complementarity to their tRNA primer are at the top of each alignment, bold and marked with a “P”. Numbers indicate how many full-length elements of that particular family in the mouse genome (mm10) had a specific PBS sequence. The most common PBS sequences are denoted “C”, and PBS sequences below are descending to less frequent variations. "Perfect" and "Common" PBS sequences are highlighted in the phylogenetic trees in Figures S1 and S2. Mismatches with the tRNA sequence are shaded in grey and black. Blue shaded nucleotide positions have been tolerant for mismatches when tested for the human immunodeficiency virus (HIV)-1, which uses tRNA Lysine^3^ to prime reverse transcription [138]. ERVs that were active in retrotransposition assays are denoted with their names on the right, next to their respective PBS sequence [114,115,131].

One would expect a trade-off between a perfect PBS for tRNA priming but susceptibility to tRF silencing. PBS sequences that allow tRNA priming but reduce tRF silencing would be most “successful” and accumulate in the pool of full-length ERVs. We collected the PBS sequences of four ERV families, whose activity has been tested in retrotransposition assays, and compared them for mismatches with the perfect PBS (Figure 2). Over time, ERVs accumulate random mutations just like any other sequence in the genome. ERVs that acquire beneficial mutations should proliferate more and dominate the sequences we find in the genome, while deleterious mutations will halt copy number increase. Strikingly, the most common PBS sequences for the examined ETn, MusD, and IAP families have mismatches with the tRNA primer and have mutations compared to a “perfect” PBS. This strongly suggests that mutations in the PBS indeed confer an advantage for the ERV, most likely because they reduce silencing by tRFs. These mutations still allow tRNA priming and replication, as elements with the most common PBS were active in retrotransposition assays (see Figure 2) [114,115,131,132]. We drew phylogenetic trees of the full-length ETn and MusD sequences to estimate whether these mutations occurred in one highly active ERV that produced high copy numbers or whether these permissive mutations got fixed in independent events (Figures S1 and S2). The phylogenetic trees of both, ETn and MusD, show perfect (0 mismatch with tRNA primer) and most common (two mismatches) PBS sequences across the entire tree, not from a single clade, indicating repeated, independent mutation events and specific mutations allowing higher copy numbers. Among highly related ERVs, such as in the top right corner of the ETn cladogram, some seem to have “reverted” from the common PBS to a perfect one (Figures S1 and S2). We would like to speculate that this is due to the tRNA primer sequence being copied and inherited with a 50:50 chance, similar to PBS propagation in other *Retroviridae*. The *Retroviridae* HIV and Moloney MLV copy the primer tRNA sequence during second strand synthesis and therefore produce dsDNA and insertions that carry their parent PBS on one strand and the tRNA copy on the other strand (Figure 1) [133,134]. In contrast, the *Pseudoviridae* copia transposon yeast 1 (Ty1) and *Metaviridae* gypsy Ty3 elements copy the PBS during reverse transcription to both strands and therefore strictly inherit their PBS sequence [135]. The PBS alignments and phylogenetic analysis of the ETnIIbeta family also revealed that ETn can switch to use a Lys^1,2^-CUU tRNA as a primer (Figure 2b and Figure S1). Indeed, a known active ETnI1 element carries a Lysine^1,2^ signature and its PBS sequence was added to the ETnIIbeta alignment for comparison (Figure 2b). Similarly, rare HIV-1 copies have been isolated that use an alternative Lysine primer [136], and MLV which usually uses tRNA^Pro1,2^ can adapt to use tRNA^Gln-GUC^ [137]. 

Of note, the majority of PBS mutations are found at certain positions (Figure 2, shaded in black). These could be mutations permissive or beneficial to retrotransposition or, alternatively, mutational hot spots during reverse transcription and DNA maintenance. DNA methylation at CpG residues often leads to mutations. These manifest as C to A and T to G changes across two adjacent bases because the cytosine of the reciprocal strand is as likely to mutate. The PBS sequences analyzed here do not follow this pattern. In addition, the most common mutations in the PBS have not accumulated so much over time but are more frequent in younger ERV copies with high percent identity between their left and right LTR (Figures S1 and S2). G to A and C to T mutations have been found in HIV-1 and are attributed to limited dCTP concentrations during reverse transcription or RNA editing of viral DNA [139]. We spot some C to T mutations, but the majority of PBS mutations cannot be explained by this mechanism. RT enzymes often misincorporate nucleotides when reading through RNA modifications, such as pseudouridine and 1-methyladenosine, that are common post-transcriptional modifications in the 3′-end of mature tRNA [140,141,142]. However, known tRNA modifications do not match the observed positions in the PBS, suggesting that these mutations are not a result of “faulty” nucleotide incorporation during reverse transcription of the tRNA primer. Instead, they were likely acquired through random mutagenesis and provided a fitness advantage to the ERV, resulting in the observed high copy numbers.

Which mutations in the PBS would benefit ERVs? Fragments of the tRF3b type, both transfected and endogenous, show all the hallmarks of miRNA silencing [22,98,101,102]. They tolerate certain mismatches with the target, for example, Lys^3^-UUU tRF3b downregulates MusD6 RNA and protein levels although it has two mismatches with its PBS [22]. For miRNA-mediated silencing, base-pairing of the “seed” nucleotides 2–7 with the target site are highly conserved and critical [143,144]. However, residues outside the seed are also important, for example, pairing with the 3′-end of miRNAs is a major determinant of AGO target specificity [145]. Similarly, miRNA-reporter assays with a tRF target site in their 3′-UTR, suggest the seed sequence is required for silencing but is not sufficient (i.e., certain mismatches outside the seed strongly reduced silencing) [98,101]. The PBS lies 2–4 nt downstream of the LTR in the 5′-UTR of ERVs, but it is likely that many of the rules for 3′-UTR targeting apply, both for canonical (seed) and non-canonical base pairing. Indeed, IAP elements with mismatches to tRFs in their PBS show higher mRNA level than IAPs with a perfect target site when released in KAP1-deficient mESC [94]. Less is known about how tRF3a fragments find and bind their targets. tRF3a^Lys3-UUU^ did not affect expression levels of ETn or MusD when transfected, but instead abrogated RT priming and were highly sensitive to mismatches with the PBS irrespective of the seed sequence [22]. This suggests that mutations in the PBS differently affect ERV expression (tRF3b) versus reverse transcription (tRF3a). Taken together, mismatches even outside the seed region relieve repression by tRFs, both for mRNA expression and during reverse transcription.

What else explains the pattern of mutations that we see at the PBS of these ERVs? If most mutations reduce silencing by tRFs, the exact positions of these mutations could be driven by whether they still allow tRNA binding and reverse transcription of the ERV. There are several lines of evidence regarding which PBS sequences allow successful tRNA priming. A number of ERVs have been cloned and found active in retrotransposition assays (Figure 2, right panel). Our sequence analysis reveals that actively transposing elements of the ETn, MusD, IAPE, and IAP families tolerate 0, 2, or 3 mutations or insertions-deletions (indels) in their PBS (Figure 2). Studies on HIV-1, which uses the same primer tRNA as ETn and MusD, lend additional insight. The structure of the HIV-1 reverse transcription initiation complex shows binding of the first 22 nt of the Lys^3^-UUU primer tRNA to the PBS [146]. However, complementarity to the first 6 nt of the PBS has been sufficient for HIV-1 priming if compensated by additional interactions outside the PBS [147]. A recent study examined spontaneous mutations in HIV-1 after clustered regularly interspaced short palindromic repeat (CRISPR)-editing of the PBS [138]. Of note, HIV-1 repression is more persistent when the PBS is targeted on the minus strand because of the asymmetrical inheritance of the PBS in *Retroviridae* [138]. When targeting the plus strand PBS, the virus quickly evolves to avoid a perfect PBS. These escapees tell us which positions in the PBS tolerate mutations during priming of reverse transcription. Remarkably, the majority of permissive HIV-1 PBS indels are at the same positions that ETn, MusD, IAPE, and IAP have accumulated mutations (blue shaded boxes, Figure 2), suggesting tRNA priming as well as evasion from targeting by tRFs shape PBS sequences in ERVs.

## 5. Outlook

Due to the high mutation rate of retroviruses but conservation of the PBS, several studies suggested that destroying the PBS is the ultimate “cure” to disable LTR-retroelements [138,148]. This strategy is promising to target infectious retroviruses like HIV in somatic tissues but would come at a high cost in mammalian stem cells that have co-opted ERVs for cellular functions. ERV-derived lncRNAs and LTR-driven gene-fusion transcripts could include a PBS sequence in their 5′-UTR which may serve to fine-tune expression of these RNAs, in analogy to miRNA target sites acting as ‘rheostats’ in the 3′-UTR of genes [149]. We would predict that PBS sequences in the 5′-UTR of LTR-driven genes are under positive selective pressure to keep a handle on this class of developmentally regulated genes. We believe PBS sequence variations and their prevalence in the pool of murine ERVs reflect the tug-of-war between ERV activity and tRF-silencing. Ultimately, rules of tRF3a and tRF3b fragments targeting the PBS in the 5′-UTR of ERVs will need to be confirmed experimentally. Many exciting questions remain. Which tissues use 3′-tRFs as a first line of defense against infectious LTR-retroviruses? Elevated Lys^3^-tRFs have been found in response to HIV-1 infection in T-cells [24]. Do 3′-tRFs regulate LTR-retroelements in other eukaryotes? What is the intersection of the tRF silencing pathway with other small RNA pathways? Can tRFs guide transcriptional silencing like other small RNA to re-establish heterochromatin at transposable elements that were released by genome-wide reprogramming? How many regulatory non-coding ERV transcripts and ERV-driven genes have retained a PBS site in their 5′-UTR and are regulated by tRFs throughout development? Comprehensive analysis of all PBS sites in the genome may provide insight into the genome-wide, regulatory networks that are driven by ERV expression and their regulation by 3′-tRFs.

## 6. Methods

Using CENSOR and REPBASE, the following sequences were compiled (Table 1), and their genomic coordinates were extracted from the RepeatMasker Library (20140131) of the mouse mm10 genome [150,151].

After reformatting to BED format, internal ERV sequences were merged to their corresponding LTR using the BEDtools merge function within 500 bases, on the same strand [152]. Sequences less than 500 bases were removed, and the remaining sequences were aligned with Muscle [153]. Sequences missing flanking LTRs were removed, and the frequency of each unique PBS variation was counted across the alignment (Figure 2). The maximum likelihood trees were made using the MEGA X program, with 500 and 300 bootstrap replicates, respectively (Figures S1 and S2) [154].

## Figures and Tables

**Table 1:** ERV sequences used in this study to compare PBS sequences and phylogenetic relationships.

Name	Genbank ID	Internal	LTR
ETnIIbeta3	AC126548	MMETN ^1^	ERVB7_1-LTR_MM
MusD6	AC124426	ERVB7_1-I_MM	ERVB7_1-LTR_MM
IAPE	AC123738	IAPEY4_I	IAPEY4_LTR
IAP	AC012382	IAPEZI	IAPLTR1a_MM

^1^ MMETn-int is the RepeatMasker alias of MMETN in REPBASE, according to Dfam.

## Supplementary Materials

The following are available online at https://www.mdpi.com/1999-4915/12/8/792/s1, Figure S1: ETnIIbeta phylogenetic tree, Figure S2: MusD phylogenetic tree.
