# Supplementary material for: Endogenous Retroviruses Walk a Fine Line between Priming and Silencing
Source: Viruses. 2020 Jul 23;12(8):792. doi: 10.3390/v12080792 (PMC7472051; doi:10.3390/v12080792)
Supplement: Supplementary file 1 [file viruses-12-00792-s001.pdf]

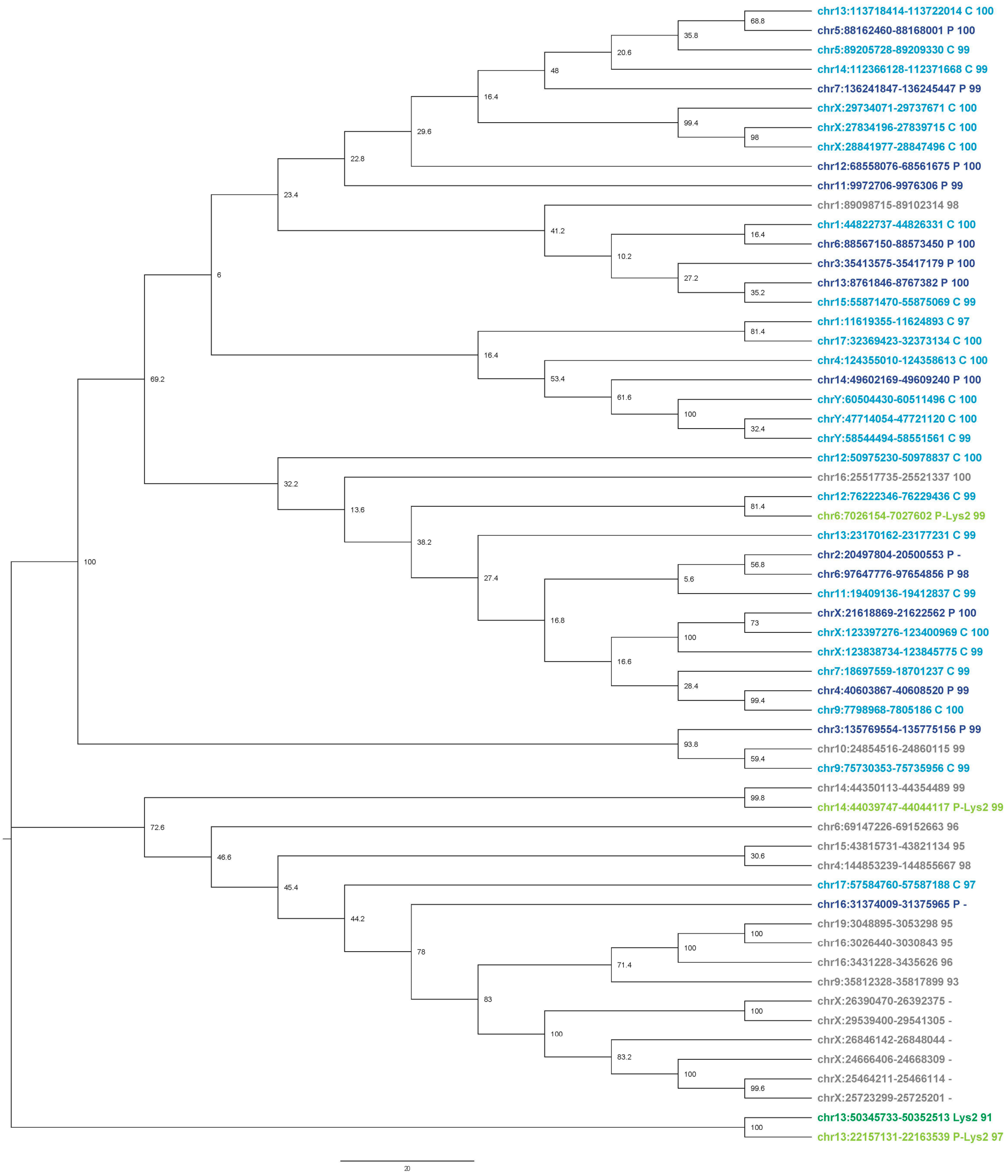

Figure S1. Maximum likelihood bootstrapped cladogram created from 59 full-length ETnIIbeta elements. Each leaf of the tree is identified with the location of the ERV, the PBS it has (see also Figure 2), and the percent identity between LTRs. "C" corresponds to the most Common PBS variant (light blue), and "P" corresponds to the PBS that has a Perfect match (navy blue) to the primer tRNA Lysine[3]. PBS that match the isodecoder Lysine[1,2] tRNA are encoded in light green for perfect complementarity (P-Lys2) or dark green for the observed Lysine [1,2] PBS sequence variant (Lys2). Bootstrap values were calculated with 500 replications. ERVs with a minus (-) instead of a percent identity did not have intact LTRs or their LTRs did not align to each other using BLAST.

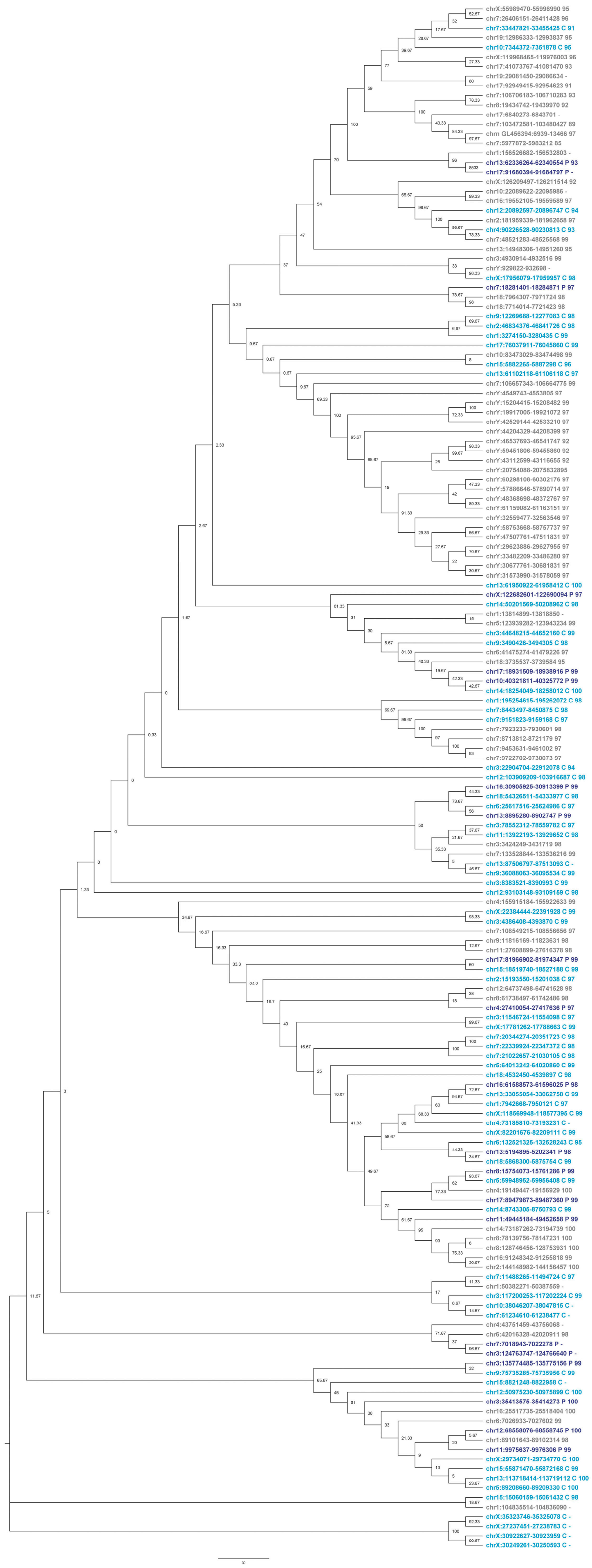

chrX:55989470-55996990 95
chr7:26406151-26411428 96
chr7:33447821-33455425 C 91
chr19:12986333-12993837 95
chr10:7344372-7351878 C 95
chrX:119968465-119976003 96
chr17:41073767-41081470 93
chr19:29081450-29086634 -
chr17:92949415-92954623 91
chr7:106706183-106710283 93
chr8:19434742-19439970 92
chr17:6840273-6843701 -
chr7:103472581-103480427 89
chrn GL456394:6939-13466 97
chr7:5977872-5983212 85
chr1:156526682-156532803 -
chr13:62336264-62340554 P 93
chr17:91680394-91684797 P -
chrX:126209497-126211514 92
chr10:22089622-22095986 -
chr16:19552105-19559589 97
chr12:20892597-20896747 C 94
chr2:181959339-181962658 97
chr4:90226528-90230813 C 93
chr7:48521283-48525568 99
chr13:14948306-14951260 95
chr3:4930914-4932516 99
chrY:929822-932698 -
chrX:17956079-17959957 C 98
chr7:18281401-18284871 P 97
chr18:7964307-7971724 98
chr18:7714014-7721423 98
chr9:12269688-12277083 C 98
chr2:46834376-46841726 C 98
chr1:3274150-3280435 C 99
chr17:76037911-76045860 C 99
chr10:83473029-83474498 99
chr15:5882265-5887298 C 96
chr13:61102118-61106118 C 97
chr7:106657343-106664775 99
chrY:4549743-4553805 97
chrY:15204415-15208482 99
chrY:19917005-19921072 97
chrY:42529144-42533210 97
chrY:44204329-44208399 97
chrY:46537693-46541747 92
chrY:59451806-59455860 92
chrY:43112599-43116655 92
chrY:20754088-2075832895
chrY:60298108-60302176 97
chrY:57886646-57890714 97
chrY:48368698-48372767 97
chrY:61159082-61163151 97
chrY:32559477-32563546 97
chrY:58753668-58757737 97
chrY:47507761-47511831 97
chrY:29623886-29627955 97
chrY:33482209-33486280 97
chrY:30677761-30681831 97
chrY:31573990-31578059 97
chr13:61950922-61958412 C 100
chrX:122682601-122690094 P 97
chr14:50201569-50208962 C 98
chr1:13814899-13818850 -
chr5:123939282-123943234 99
chr3:44648215-44652160 C 99
chr9:3490426-3494305 C 98
chr6:41475274-41479226 97
chr18:3735537-3739584 95
chr17:18931509-18938916 P 99
chr10:40321811-40325772 P 99
chr14:18254049-18258012 C 100
chr1:195254615-195262072 C 98
chr7:8443497-8450875 C 98
chr7:9151823-9159168 C 97
chr7:7923233-7930601 98
chr7:8713812-8721179 97
chr7:9453631-9461002 97
chr7:9722702-9730073 97
chr3:22904704-22912078 C 94
chr12:103909209-103916687 C 98
chr16:30905925-30913399 P 99
chr18:54326511-54333977 C 98
chr6:25617516-25624986 C 97
chr13:8895280-8902747 P 99
chr3:78552312-78559782 C 97
chr11:13922193-13929652 C 98
chr3:3424249-3431719 98
chr7:133528844-133536216 99
chr13:87506797-87513093 C -
chr9:36088063-36095534 C 99
chr3:8383521-8390993 C 99
chr12:93103148-93109159 C 98
chr4:155915184-155922633 99
chrX:22384444-22391928 C 99
chr3:4386408-4393870 C 99
chr7:108549215-108556656 97
chr9:11816169-11823631 98
chr11:27608899-27616378 98
chr17:81966902-81974347 P 99
chr15:18519740-18527188 C 99
chr2:15193550-15201038 C 97
chr12:64737498-64741528 98
chr8:61738497-61742486 98
chr4:27410054-27417636 P 97
chr3:11546724-11554098 C 97
chrX:17781262-17788663 C 99
chr7:20344274-20351723 C 98
chr7:22339924-22347372 C 98
chr7:21022657-21030105 C 98
chr5:64013242-64020860 C 99
chr18:4532450-4539897 C 98
chr16:61588573-61596025 P 98
chr13:33055054-33062758 C 99
chr1:7942668-7950121 C 97
chrX:118569948-118577395 C 99
chr4:73185810-73193231 C -
chrX:82201676-82209111 C 99
chr6:132521325-132528243 C 95
chr13:5194895-5202341 P 98
chr18:5868300-5875754 C 99
chr8:15754073-15761286 P 99
chr5:59948952-59956408 C 99
chr4:19149447-19156929 100
chr17:89479873-89487360 P 99
chr14:8743305-8750793 C 99
chr11:49445184-49452658 P 99
chr14:73187262-73194739 100
chr8:78139756-78147231 100
chr8:128746456-128753931 100
chr16:91248342-91255818 99
chr2:144148982-144156457 100
chr7:11488265-11494724 C 97
chr1:50382271-50387559 -
chr3:117200253-117202224 C 99
chr10:38046207-38047815 C -
chr7:61234610-61238477 C -
chr4:43751459-43756068 -
chr6:42016328-42020911 98
chr7:7018943-7022278 P -
chr3:124763747-124766640 P -
chr3:135774485-135775156 P 99
chr9:75735285-75735956 C 99
chr15:8821248-8822958 C -
chr12:50975230-50975899 C 100
chr3:35413575-35414273 P 100
chr16:25517735-25518404 100
chr6:7026933-7027602 99
chr12:68558076-68558745 P 100
chr1:89101643-89102314 98
chr11:9975637-9976306 P 99
chrX:29734071-29734770 C 100
chr15:55871470-55872168 C 99
chr13:113718414-113719112 C 100
chr5:89208660-89209330 C 100
chr15:15060159-15061432 C 98
chr1:104835514-104836090 -
chrX:35323746-35325078 C -
chrX:27237451-27238783 C -
chrX:30922627-30923959 C -
chrX:30249261-30250593 C -

Figure S2. Maximum likelihood bootstrapped cladogram of 161 full-length MusD elements. Each node of the tree has a bootstrap value calculated from 300 replications. Each leaf of the tree has the location of a MusD element, the code for the PBS variant that it has (see also Figure 2), and the percent identity between LTRs of each element. The Common (C) code in light blue corresponds to the most prevalent PBS variant, and the P code in navy blue corresponds to the PBS that has a Perfect match to the Lysine[3] tRNA primer. ERVs with a minus (-) instead of a percent identity did not have intact LTRs or their LTRs did not align to each other using BLAST.
